# Association of physical activity and vitamin D deficiency with cognitive impairment in older adults: a population based cross-sectional analysis

**DOI:** 10.3389/fnut.2024.1390903

**Published:** 2024-05-01

**Authors:** Jing Guo, Hongfei Mo, Longfei Zuo, Xu Zhang

**Affiliations:** ^1^National Engineering Laboratory for Internet Medical Systems and Applications, The First Affiliated Hospital of Zhengzhou University, Zhengzhou, Henan, China; ^2^Department of Kinesiology, School of Physical Education (Main Campus), Zhengzhou University, Zhengzhou, Henan, China; ^3^Department of Nutrition and Food Hygiene, College of Public Health, Zhengzhou University, Zhengzhou, Henan, China; ^4^Synergetic Innovation Center of Kinesis and Health, School of Physical Education (Main Campus), Zhengzhou University, Zhengzhou, Henan, China; ^5^Department of Children Adolescence and Woman Health, College of Public Health, Zhengzhou University, Zhengzhou, Henan, China

**Keywords:** cognitive impairment, elderly population, physical activity, vitamin D deficiency, cross-sectional analysis

## Abstract

**Objectives:**

The global aging situation is becoming increasingly critical and cognitive impairment in the elderly has become a public health burden of concern. Physical activity (PA) and vitamin D may play a key role in improving cognitive impairment. However, little studies have examined the interaction between these two. The purpose of this study was to assess the association of PA and vitamin D with cognitive impairment in older adults, as well as the interactions of PA and vitamin D.

**Materials and methods:**

This study was conducted by multi-stage random sampling of elderly people ≥60 years old, and a total sample of 2,492 (1,207 male and 1,285 female, mean age of 69.41 ± 6.75 years) with complete data was included in the analysis. PA was assessed by the Global Physical Activity Questionnaire, and < 600 MET-min/week was used as the division criteria. Serum vitamin D was measured by high-performance liquid chromatography tandem mass spectrometry, and 25-hydroxyvitamin D2/D3 concentration < 20 ng/mL was used as a vitamin D deficiency criterion. Cognitive function was assessed by three subtests: the Consortium to Establish a Registry for Alzheimer’s disease word learning test (CERAD-WL) for immediate and delayed learning, the Animal Fluency Test (AFT) for verbal fluency; and the Digit Symbol Substitution Test (DSST) for information processing speed and switching attention. All three subtests were scored at less than the lowest quartile of the score as a criterion for cognitive impairment. Statistical analysis was performed using SPSS for chi-square test, rank sum test, interaction analysis, subgroup analysis, and regression analysis.

**Results:**

Lower level of PA is associated with higher odds of cognitive impairment (CERAD W-L: OR = 1.596, 95% CI: 1.338–1.905, *p* < 0.001; AFT: OR = 1.833, 95% CI: 1.534–2.190, *p* < 0.001; DSST: OR = 1.936, 95% CI: 1.609–2.329, *p* < 0.001). Vitamin D deficiency has significant effects in AFT (OR = 1.322, 95% CI: 1.103–1.584, *p* = 0.003) and DSST (OR = 1.619, 95% CI: 1.345–1.948, *p* < 0.001). After adjusted for covariates, PA and vitamin D have multiplicative interaction on AFT (OR = 0.662, 95% CI: 0.448–0.977, *p* = 0.038) and DSST (OR = 0.775, 95% CI: 0.363–0.868, *p* = 0.009). The interaction between PA and vitamin D was not significant in the CERAD W-L (OR = 0.757, 95% CI: 0.508–1.128, *p* = 0.172).

**Conclusion:**

The results showed that lower level of PA and vitamin D deficiency were associated with higher odds of cognitive impairment in the elderly population and that there was a multiplicative interaction between PA and vitamin D on cognitive function, with a significant effect of vitamin D on cognitive impairment in high PA conditions.

## 1 Background

The global aging situation is becoming increasingly critical, leading to a rapidly deteriorating situation of cognitive impairment in the elderly population. The World Health Organization (WHO) has previously estimated that the global population with cognitive impairment will increase acutely from 55 million in 2019 to 139 million by 2050 (1). Cognitive impairment is a group of severe learning and memory deficits due to the abnormalities in the processing of higher brain intelligence related to learning, memory, and thinking judgments (2). The prevalence of cognitive impairment is significantly higher in the elderly population than in other age groups (3). The occurrence of cognitive impairment in the elderly population has a significant economic impact on families and communities, increasing social exclusion and individual stigma. Cognitive functioning is also directly related to quality of life, interpersonal relationships, and self-independence in this population, which together constitute a significant public health burden (4). However, there are currently very limited treatments for cognitive impairment and no targeted medications (5). Therefore, it is particularly important to uncover potential risk factors and protective factors for cognitive impairment in the elderly population to guide the development of prevention strategies.

Evidence suggested that daily physical activity (PA) may be an important intervention for cognitive decline in older adults (6). PAs are performed by skeletal muscles for any movement that requires energy expenditure, where moderate-to-vigorous physical activity (MVPA) are PAs greater than 3 metabolic equivalents (METs) and include activities such as brisk walking, cycling, and conditioning exercise (7). MVPA can prevent a variety of chronic conditions including cognitive impairment in the elderly, and studies have found that PA can reduce the risk of dementia and Alzheimer’s disease by 28 and 45%, respectively (8). Vitamin D is another factor that may significantly affect cognitive conditions in elderly population. Vitamin D deficiency is a global public health burden that afflicts over 1 billion people worldwide (9), which consequences should not be underestimated. Vitamin D can affect neurocognition through multiple pathways, including induction of neuroprotection, regulation of oxidative stress, modulation of calcium homeostasis, and inhibition of inflammatory processes (10). Previous studies have found that Vitamin D deficiency may be more common in older populations and may contribute to cognitive decline, dementia, and Alzheimer’s disease (11).

Physical activity and vitamin D have long been interrelated, and evidence have suggested that both PA and vitamin D have positive interactive effects on numerous psychiatric outcomes (12). However, the relationship between vitamin D, PA, and cognitive impairment, and the interactive effects of vitamin D and PA on cognitive impairment in elderly population are yet not fully understood. Older adults may have difficulty participating in regular PA due to decreased mobility, lack of safe outdoor facilities, and other health issues. At the same time, cognitive decline may also lead to decreased PA and reduced time spent outdoors. In addition, vitamin D deficiency has been shown to play a role in age-related cognitive decline (13), further complicating its relationship with PA and cognitive impairment. Given these challenges, further research is needed to understand the relationship between vitamin D deficiency, PA, and cognitive function in elderly population. Therefore, in this study, we analyzed the effects of PA and vitamin D deficiency on cognitive impairment in a national representative elderly population, respectively, and additionally analyzed the interaction between PA and vitamin D deficiency on cognitive impairment. These analyses will allow us to develop evidence-based interventions to promote brain health and physical health in elderly population.

## 2 Materials and methods

### 2.1 Study population

**Figure 1:**
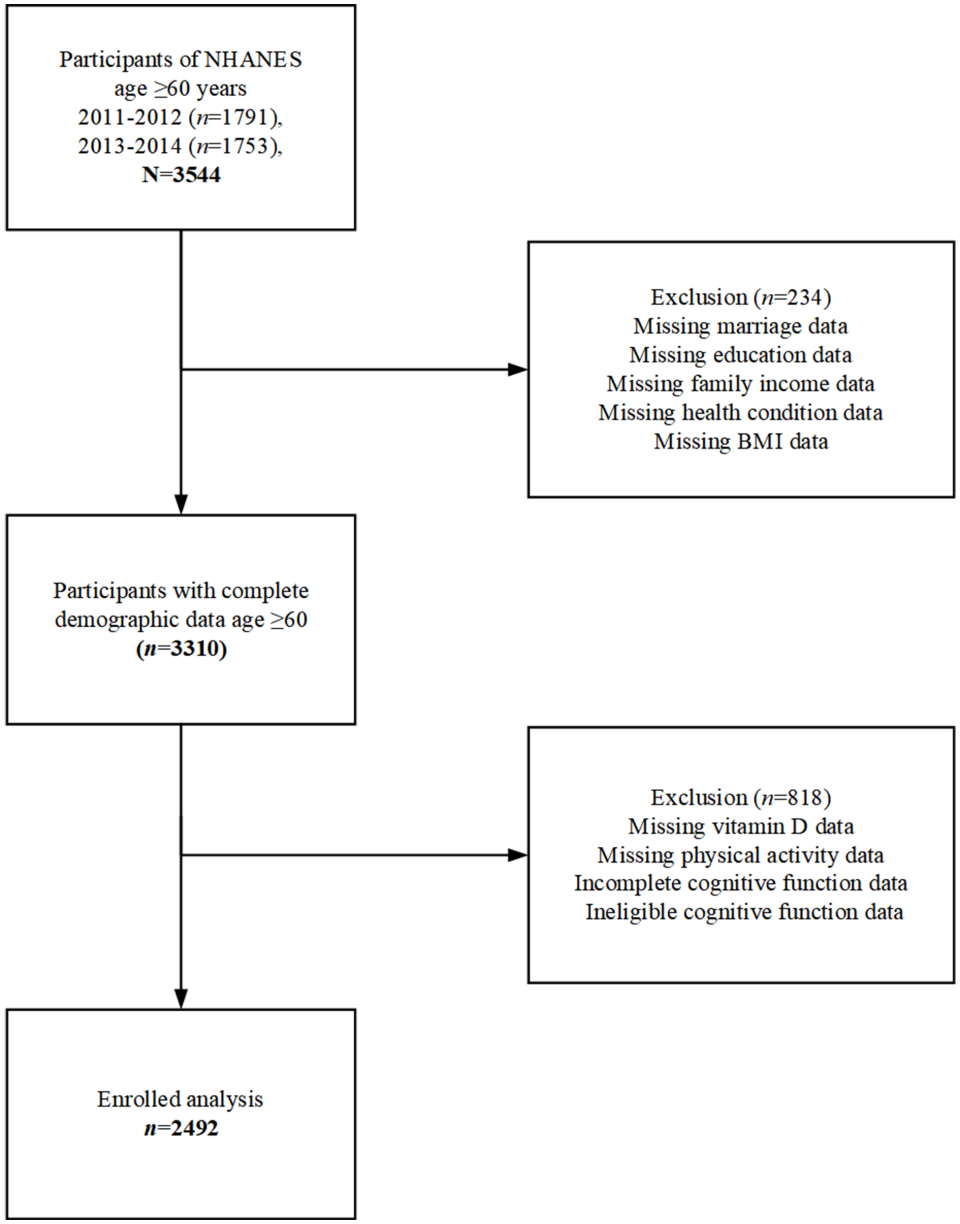
Flow chart of the selection process for selecting eligible participants.

In this study, we analyzed data from the National Health and Nutrition Examination Survey (NHANES), a population-based cross-sectional survey designed to collect information on the health and nutritional status of an all-age population in the United States (U.S.). The survey is conducted on a 2-year cycle and includes household interviews and health assessments. The NHANES protocol and secondary analysis of the data were approved by the National Center for Health Statistics (NCHS) Ethics Review Board, and all adult participants signed a written notice of consent. In this study, we extracted data from two survey cycles, 2011–2012 and 2013–2014. Participants aged 60 years and older, with complete demographic information, who completed the cognitive function assessment tests, serum vitamin D examinations, and the PA questionnaire were included in this study. We screened a total of 3,544 participants aged 60 years and older and excluded those with missing data on demographic characteristics (*n* = 234), incomplete or ineligible data on cognitive function, missing vitamin D data, and missing PA data (*n* = 818). Finally, a total of 2,492 participants were included in the analysis of this study (See Figure 1).

### 2.2 Cognitive function assessments

In this study, cognitive function is measured by the following three tests: word learning and recall modules from the Consortium to Establish a Registry for Alzheimer’s disease (CERAD), the Animal Fluency test (AFT), and the Digit Symbol Substitution test (DSST). The three tests above assess three different aspects of cognitive function.

The CERAD Word Learning subtest (CERAD W-L) consists of three consecutive learning trials and a delayed recall to assess the ability to learn new words and recall words, which has been used in major epidemiological studies in different ethnic and cultural communities (14). During the learning trials, participants first engage in learning 10 unrelated words and after recall as many as possible immediately after learning them. Delayed recall will be done after AFT and DSST are finished.

The AFT test evaluates categorical verbal fluency, whose scores have been shown to discriminate the cognitive function between the cognitive impairment and normal cognitive functioning (15). There will be a practice test before AFT, participants who finished the pretest will continue the AFT. The AFT requires participants to name as many as animals as possible in 1 min. The score of AFT is the sum of the names of the animals in 1 min.

The DSST test is a well-established measure to assess executive function and processing speed, which has been used in epidemiological and clinical studies (16). According to the NHANES, participants who did not finished the practice test will not continue. The DSST asked participants to match the numbers and the symbols; the score is the total number of numbers correctly matched with their corresponding symbols for 133 numbers in 2 min.

In the present study, we referred to the classification method of previous studies and used the lowest quartile of participant scores on the CERAD W-L, AFT, and DSST tests, respectively, as cut-off points (17). Participants with test scores below or equal to the cut-off point were included in the Cognitive Impairment group, whereas participants with test scores above the cut-off point were included in the Normal Cognition group. For the processing of the data, we did not sum or take the maximum of the scores of the three tests but exhibited them separately in the subsequent table.

### 2.3 Physical activity assessments

In this study, PA was assessed through interview that was based on the Global Physical Activity Questionnaire (GPAQ) conducted at participants’ home by trained technicians using the Computer Assisted Personal Interview (CAPI) system. According to the questions related to daily activities and leisure time activities, PAs were classified as work physical activity (WPA), transportation physical activity (TPA), and recreational physical activity (RPA) according to their different purposes or intensity of implementation by the participants. WPA refers to the things that must be done, such as household chores, yard work, and paid or unpaid work. Vigorous WPA causes a large increase in breathing or heart rate and lasts for at least 10 min continuously, such as lifting or carrying heavy loads. Activities like walking at a fast pace or lifting light loads for at least 10 min that causes a slight increase in breathing or heart rate are considered moderate WPA. TPA is defined as walking or biking for at least 10 min continuously on the way to school, shopping, or work. The RPA excludes work and transportation activities already mentioned, and high-intensity sports, fitness, or recreational activities that cause a large increase in breathing or heart rate are classified as vigorous RPA, such as running and basketball, and activities that cause a small increase in heart rate or breathing, such as biking and swimming, are classified as moderate RPA. During this process, participants answered what types of PAs they had performed in the past week and the duration of time they performed that type of PA. According to the NHANES metabolic equivalent score recommendations: eight METs for vigorous WPA, four METs for moderate WPA, four METs for TPA, eight METs for vigorous RPA, and four METs for moderate RPA. In this study, we assessed PA by multiplying the “duration of different types of activities (in minutes)” by the “intensity (in METs)” score of participants’ performance in the past week. According to the U.S. Physical Activity Guidelines, high-intensity PA is defined as ≥600 MET-min/week and low-intensity PA is defined as <600 MET-min/week (18). Therefore, we used 600 MET-min/week as the cut-off point for PA in this study.

### 2.4 Vitamin D assessment

Vitamin D level was measured through methods from Centers for Disease Control (CDC): the test principle used high-performance liquid chromatography tandem mass spectrometry (HPLC-MS/MS) to quantitatively detect 25-hydroxyvitamin D3 (25OHD3), 3-epi-25-hydroxyvitamin D3 (epi-25OHD3), and 25-hydroxyvitamin D2 (25OHD2) in human serum. In this study, we used the concentration of serum 25-hydroxyvitamin D2 plus serum 25-hydroxyvitamin D3, collectively referred to as 25(OH)D, to assess the participants’ vitamin D level. According to the Endocrine Society, 20 ng/mL was used as a classification criterion for vitamin D level in adults (9). Therefore, in this study, 25(OH)D < 20 ng/mL was classed as vitamin D deficiency, and 25(OH)D ≥ 20 ng/mL was classed as moderate to excessive level (no deficiency).

### 2.5 Covariates

The covariates in this study contained gender, age, race, season of exam, education level, marital status, income to poverty, body mass index (BMI), and recent health status. Each of these covariates may have an impact on the outcomes of this study. Age was categorized into three age groups: 60–69, 70–79, and ≥ 80 years. Race was divided into five categories: Hispanic, non-Hispanic White, non-Hispanic Black, non-Hispanic Asian, and Other. The season of exam consisted of two periods: November 1 to April 30 and May 1 to October 31. Education level was divided into three categories: below high school, high school, and above high school. Marital status was categorized as “married/living with partner” and “married living alone (widowed, divorced, separated)/never married.” In this study, income to poverty was used to indicate the income status of participants, which was divided into two categories: impoverished and moderate income. Income to poverty <1.3 was defined as impoverished, and ≥ 1.3 as moderate income (19). BMI was classified into three categories, with <25 kg/m^2^, 25 to <30 kg/m^2^, and ≥30 kg/m^2^ indicating low and standard weight (here we combined the classifications because the number of underweights with BMI <19 is so small that may trigger bias), overweight and obese (20). According to the NHANES classification, recent health status was classified into five categories: excellent, very good, good, fair, and poor.

### 2.6 Statistical analysis

We first screened the raw data using Microsoft Excel 2010 to exclude participants who did not meet the inclusion criteria, which included missing demographic information, missing recent health status or BMI data (*n* = 234), and participants who did not complete the cognitive function test or missing serum vitamin D or PA data (*n* = 818). Afterward, we performed chi-squared tests to initially screen for covariates with statistically significant differences between the Normal Cognition group and Cognitive Impairment group in the CERAD W-L/AFT/DSST tests, respectively. A-entry = 0.05 and a-exit = 0.10 were used to select and exclude independent variables.

We then performed binary logistic regression analyses to estimate the association between PA and cognitive function as reflected by CERAD W-L/AFT/DSST test results, and the association between cognitive function as reflected by vitamin D status CERAD W-L/AFT/DSST test results, respectively. We also developed binary stepwise regression models with vitamin D as the independent variable and PA as the dependent variable, and divided PA into “High PA group” and “Low PA group” to analyze the association between vitamin D status and PA after excluding the effects of confounding variables. In this stepwise regression analysis, three models were developed: Model 1: original model without adjusting for any variables; Model 2: adjusted for age, gender, race, education level, marital status, income to poverty, recent health status and BMI; Model 3: adjusted for the independent variables in Model 2 plus test results from CERAD W-L/AFT/DSST tests.

Finally, we constructed binary stepwise logistic regression models for interaction analyses, which were used to analyze the interaction relationship between PA and VD in their association with cognitive function. To analyze the effect of vitamin D on cognitive function as reflected by the recognition CERAD W-L/AFT/DSST tests in both high and low PA conditions, respectively. We have set two subgroups, “high PA group” and “low PA group,” with vitamin D status as the independent variable and cognitive function reflected by CERAD W-L/AFT/DSST test as the dependent variable and analyzed the association between vitamin D and cognitive function, respectively, in the subgroups. To exclude the effect of confounding variables, we sequentially included statistically significant covariates from chi-square tests (*p* < 0.05) in the stepwise regression model. In the model setting, we considered the possible significant impact of recent health status on cognitive function (21). This led to the development of three models: Model 4: original model without adjusting for any variables; Model 5: adjusted for age, gender, race, education level, marital status, income to poverty; Model 6: adjusted for the independent variables in Model 5 plus recent health status. In this study, all data were analyzed using the Statistical Package for Social Sciences (SPSS) version 28.0. Visualization was achieved through GraphPad Prism 8th Generation. *p* values less than 0.05 were considered statistically significant (two-sided test).

## 3 Results

### 3.1 Demographic characteristics

A total of 2,492 participants aged 60 years and older who completed cognitive function tests and had complete demographic, physical activity, and vitamin D data were included in this study. The mean age of the participants was 69.41 ± 6.75 years, of which 1,207 (48.4%) were male and 1,285 (51.6%) were female.

On the CERAD W-L, differences in age, gender, education level, income to poverty, and recent health status between the Normal Cognitive Group and the Cognitive Impairment Group were statistically significant (all *p* < 0.001), as were differences in race and marital status (*p* = 0.002, *p* = 0.029), while differences in season of exam and BMI were not significant (*p* = 0.768, *p* = 0.116).

On the AFT, differences in age, race, education level, income to poverty, and recent health status between the Normal Cognitive Group and the Cognitive Impairment Group were statistically significant (all *p* < 0.001), as were differences in marital status (*p* = 0.004), whereas differences in gender, season of exam, and BMI were not significant (*p* = 0.378, *p* = 0.569, *p* = 0.116).

On the DSST, differences in age, gender, race, education level, marital status, income to poverty, and recent health status between the Normal Cognitive Group and the Cognitive Impairment Group were statistically significant (all *p* < 0.001), while differences in season of exam and BMI were not significant (*p* = 0.068, *p* = 0.616). (See Table 1).

**Table 1:** Demographic characteristics of participants age ≥ 60 from NHANES 2011 to 2014 by cognitive function.

Characters, *n*%	Number of subjects (*N*)		CERAD W-L					Animal Fluency Test					Digit Symbol Substitution Test				
			Normal cognition		Cognitive impairment		*p*-value	Normal cognition		Cognitive impairment		*p*-value	Normal cognition		Cognitive impairment		*p*-value
Age group							<0.001					<0.001					<0.001
60–69 years	1,360	(54.6)	1,087	(79.9)	273	(20.1)		1,039	(76.4)	321	(23.6)		1,084	(79.7)	276	(20.3)	
70–79 years	734	(29.5)	499	(68.0)	235	(32.0)		506	(68.9)	228	(31.1)		514	(70.0)	220	(30.0)	
≥80 years	398	(16.0)	200	(50.3)	198	(49.7)		239	(60.1)	159	(39.9)		253	(63.6)	145	(36.4)	
Gender							<0.001					0.378					<0.001
Male	1,207	(48.4)	787	(65.2)	420	(34.8)		874	(72.4)	333	(27.6)		851	(70.5)	356	(29.5)	
Female	1,285	(51.6)	999	(77.7)	286	(22.3)		910	(70.8)	375	(29.2)		1,000	(77.8)	285	(22.2)	
Race							0.002					<0.001					<0.001
Hispanic	452	(18.1)	292	(64.6)	160	(35.4)		312	(69.0)	140	(31.0)		253	(56.0)	199	(44.0)	
Non-Hispanic White	1,247	(50.0)	914	(73.3)	333	(26.7)		991	(79.5)	256	(20.5)		1,046	(84.1)	198	(15.9)	
Non-Hispanic Black	568	(22.8)	407	(71.7)	161	(28.3)		339	(59.7)	229	(40.3)		356	(62.7)	212	(37.3)	
Non-Hispanic Asian	191	(7.7)	149	(78.0)	42	(22.0)		118	(61.8)	73	(38.2)		164	(85.9)	27	(14.1)	
Other	34	(1.4)	24	(70.6)	10	(29.4)		24	(70.6)	10	(29.4)		29	(85.3)	5	(14.7)	
Season of exam							0.768					0.569					0.068
November–April	1,134	(45.4)	808	(71.4)	324	(28.6)		804	(71.0)	328	(29.0)		821	(72.5)	331	(27.5)	
May–October	1,362	(54.6)	978	(71.9)	382	(28.1)		980	(72.1)	380	(27.9)		1,030	(75.7)	330	(24.3)	
Education level							<0.001					<0.001					<0.001
Below high school	595	(23.9)	322	(54.1)	273	(45.9)		332	(55.8)	263	(44.2)		247	(41.5)	348	(58.5)	
High school	589	(23.6)	418	(71.0)	171	(29.0)		391	(66.4)	198	(33.6)		437	(74.2)	152	(25.8)	
Above high school	1,308	(52.5)	1,046	(80.0)	262	(20.0)		1,061	(81.1)	247	(18.9)		1,167	(89.2)	141	(10.8)	
Marital status							0.029					0.004					<0.001
Married/living with partner	1,441	(57.8)	1,057	(73.4)	384	(26.6)		1,064	(73.8)	377	(26.2)		1,127	(78.2)	314	(21.8)	
Married living alone/never married	1,051	(42.2)	729	(69.4)	322	(30.6)		720	(68.5)	331	(31.5)		724	(68.9)	327	(31.1)	
Income to poverty							<0.001					<0.001					<0.001
Impoverished	728	(29.2)	446	(61.3)	282	(38.7)		443	(60.9)	285	(39.1)		412	(56.6)	316	(43.4)	
Moderate income	1764	(70.8)	1,340	(76.0)	424	(24.0)		1,341	(76.0)	423	(24.0)		1,439	(81.6)	325	(18.4)	
Body mass index							0.116					0.116					0.616
<25 kg/m^2^	663	(26.6)	461	(69.5)	202	(30.5)		454	(68.5)	209	(31.5)		483	(72.9)	180	(27.1)	
25–<30 kg/m^2^	880	(35.3)	623	(70.8)	257	(29.2)		640	(72.7)	240	(27.3)		659	(74.9)	221	(25.1)	
≥30 kg/m^2^	949	(38.1)	702	(74.0)	247	(26.0)		690	(72.7)	259	(27.3)		709	(74.7)	240	(25.3)	
Recent health status							<0.001					<0.001					<0.001
Excellent	190	(7.6)	147	(77.4)	43	(22.6)		148	(77.9)	42	(22.1)		156	(82.1)	34	(17.9)	
Very good	642	(25.8)	496	(77.3)	146	(22.7)		529	(82.4)	113	(17.6)		548	(85.4)	94	(14.6)	
Good	989	(39.7)	730	(73.8)	259	(26.2)		708	(71.6)	281	(28.4)		773	(78.2)	216	(21.8)	
Fair	556	(22.3)	353	(63.5)	203	(36.5)		338	(60.8)	218	(39.2)		319	(57.4)	237	(42.6)	
Poor	115	(4.6)	60	(52.2)	55	(47.8)		61	(53.0)	54	(47.0)		55	(47.8)	60	(52.2)	

### 3.2 Association of PA and cognitive impairment

**Figure 2:**
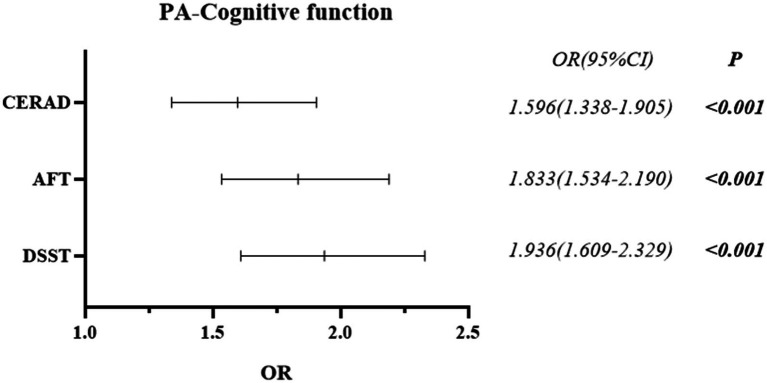
Forest plot of the *post-hoc* relationship between cognitive function and physical activity.

The differences in PA between the Normal Cognitive Group and the Cognitive Impairment group were statistically significant in all three cognitive function tests of CERAD W-L/AFT/DSST (all *p* < 0.001) (See Table 2). Cognitive impairment was more prevalent in low PA than in high PA. Binary logistic regression analysis revealed that lower levels of PA may be associated with higher odds of cognitive impairment. Compared with higher PA, lower PA was associated with 59.6% higher odds in CERAD W-L (OR = 1.596, 95% CI: 1.338–1.905), 83.3% in AFT (OR = 1.833, 95% CI: 1.534–2.190), and 93.6% in DSST (OR = 1.936, 95% CI: 1.609–2.329) (See Figure 2).

**Table 2:** Cognitive function characteristics of NHANES 2011 to 2014 adults age ≥ 60 by physical activity.

	High physical activity		Low physical activity		OR	95% CI	*p*-value
	*n* = 1,209		*n* = 1,283				
CERAD W-L					1.596	1.338–1.905	<0.001
Normal cognition	925	(76.5)	861	(67.1)			
Cognitive impairment	284	(23.5)	422	(32.9)			
Animal Fluency Test					1.833	1.534–2.190	<0.001
Normal cognition	941	(77.8)	843	(65.7)			
Cognitive impairment	268	(22.2)	440	(34.3)			
Digit Symbol Substitution Test					1.936	1.609–2.329	<0.001
Normal cognition	975	(80.6)	876	(68.3)			
Cognitive impairment	234	(19.4)	407	(31.7)			

### 3.3 Association of vitamin D and cognitive impairment

**Figure 3:**
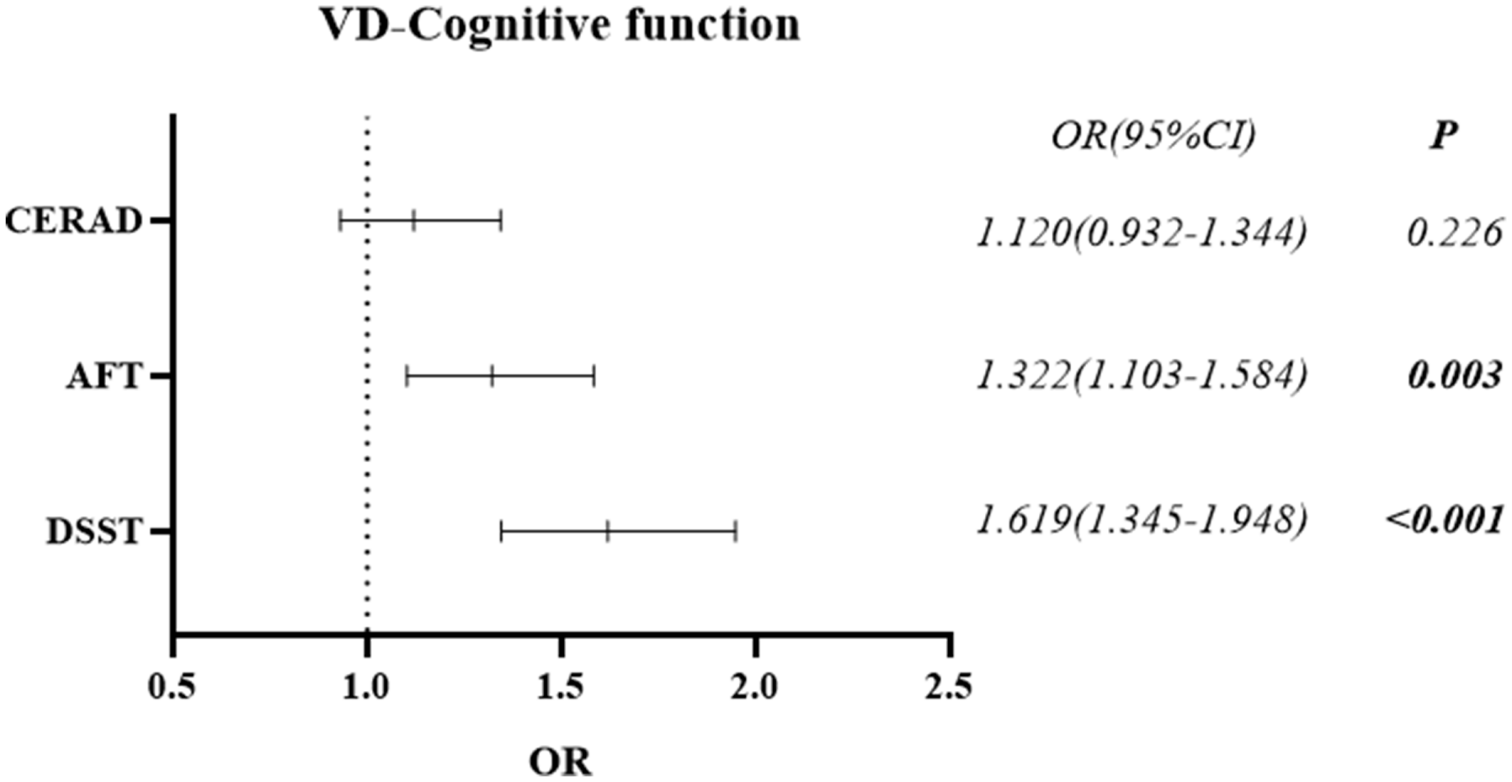
Forest plot of the *post-hoc* relationship between cognitive function and vitamin D.

The differences in vitamin D between the Normal Cognitive Group and the Cognitive Impairment group were statistically significant in the cognitive function tests of AFT and DSST (*p* = 0.003, *p* < 0.001), but not in CERAD W-L (*p* = 0.226) (See Table 3). Binary logistic regression analysis revealed that vitamin D deficiency may be associated with higher odds of cognitive impairment. Compared with the non-deficiencies, vitamin D deficiency was associated with 32.2% higher odds in AFT (OR = 1.322, 95% CI: 1.103–1.584), and 61.9% in DSST (OR = 1.619, 95% CI: 1.345–1.948) (See Figure 3).

**Table 3:** Cognitive function characteristics of NHANES 2011–2014 adults age ≥ 60 by vitamin D.

	VD deficiency		VD non-deficiency		OR	95% CI	*p*-value
	*n* = 844		*n* = 1,648				
CERAD W-L					1.120	0.932–1.344	0.226
Normal cognition	592	(70.1)	1,194	(72.5)			
Cognitive impairment	252	(29.9)	454	(27.5)			
Animal Fluency Test					1.322	1.103–1.584	0.003
Normal cognition	572	(67.8)	1,212	(73.5)			
Cognitive impairment	272	(32.2)	436	(26.6)			
Digit Symbol Substitution Test					1.619	1.345–1.948	<0.001
Normal cognition	574	(68.0)	1,277	(77.5)			
Cognitive impairment	270	(32.0)	371	(22.5)			

### 3.4 Association of PA and vitamin D

In analysis for the association of PA and vitamin D, age, gender, race, education level, marital status, income to poverty, recent health status and BMI, as well as test results from CERAD W-L/AFT/DSST were included in the regression models. Model 1 (without excluding any confounders) showed an odds ratio (OR) of 1.300 (95% CI: 1.100–1.536) (*p* = 0.002) for the association of PA and vitamin D; Model 2 (adjusted for variables of age, gender, race, education level, marital status, income to poverty, recent health status, and BMI) showed OR = 1.255 (95% CI: 1.046–1.506) (*p* = 0.015), Model 3 (adjusted for test results from CERAD W-L/AFT/DSST) showed OR = 1.232 (95% CI: 1.026–1.480) (*p* = 0.025). The results suggest that lower levels of PA is associated with higher odds of vitamin D deficiency after adjusted for confounders. Participants with low PA are 23.2% more likely to be vitamin D deficient than those who attend greater intensity PA (See Table 4).

**Table 4:** Association of PA and vitamin D deficiency in NHANES adults age ≥ 60.

Model	*b*	SE	Wald	*p*-value	OR(95%CI)
Model 1^a^	0.262	0.085	9.522	0.002	1.300 (1.100–1.536)
Model 2^b^	0.227	0.093	5.973	0.015	1.255 (1.046–1.506)
Model 3^c^	0.209	0.093	4.999	0.025	1.232 (1.026–1.480)

^a^Original model without adjusting for any variables. ^b^Adjusted for age, gender, race, education level, marital status, income to poverty, recent health status, and BMI. ^c^Adjusted for the independent variables in Model 2 plus test results from CERAD W-L/AFT/DSST.

### 3.5 Interaction association of PA and vitamin D with cognitive impairment

First, we explored the multiplicative interaction of PA and vitamin D, with cognitive impairment screened by CERAD W-L/AFT/DSST, respectively. In these analyses, the statistically significant covariates from the univariate analysis: age, gender, race, education level, marital status, income to poverty, and recent health status, was included in regression models. Model 4 (without excluding any confounding variables) showed that PA and vitamin D, on all three tests of CERAD W-L (*p* = 0.046), AFT (*p* = 0.007), and DSST (*p* = 0.001), had significant multiplicative interaction on cognitive impairment. Model 5 (which included age, gender, race, education level, marital status, and income to poverty as confounding variables in the regression analysis) showed that PA and vitamin D, on the AFT (*p* = 0.032) and DSST (*p* = 0.008), had a significant multiplicative interaction on cognitive impairment, while no statistically significant difference was observed in CERAD W-L (*p* = 0.154). Model 6 (based on model 5 with additional exclusion of recent health status) showed that PA and vitamin D, on the AFT (*p* = 0.038) and DSST (*p* = 0.009) tests, had a significant multiplicative interaction on cognitive impairment, while no statistically significant difference was observed in CERAD W-L (*p* = 0.172). The results showed that there was a significant multiplicative interaction between PA and vitamin D on cognitive impairment in AFT and DSST, especially in DSST (See Table 5).

**Table 5:** Multiplicative interaction association of vitamin D plus physical activity and cognitive function in NHANES 2011 to 2014 participants age ≥ 60 years.

	OR	95% CI	*p* for interaction	High physical activity			Low physical activity		
				OR (95% CI)		*p*-value	OR (95% CI)		*p*-value
CERAD W-L									
Model 4^d^	0.686	0.473–0.994	0.046	1.352	1.021–1.791	0.035	0.928	0.728–1.182	0.544
Model 5^e^	0.749	0.503–1.115.	0.154	1.233	0.901–1.689	0.191	0.934	0.713–1.223	0.618
Model 6^f^	0.757	0.508–1.128	0.172	1.192	0.869–1.635	0.276	0.904	0.689–1.187	0.468
Animal Fluency Test									
Model 4^d^	0.602	0.416–0.872	0.007	1.722	1.298–2.284	<0.001	1.037	0.817–1.317	0.736
Model 5^e^	0.655	0.444–0.964	0.032	1.517	1.113–2.066	0.008	1.073	0.828–1.390	0.596
Model 6^f^	0.662	0.448–0.977	0.038	1.456	1.067–1.986	0.018	1.029	0.792–1.337	0.831
Digit Symbol Substitution Test									
Model 4^d^	0.528	0.361–0.773	0.001	2.296	1.713–3.079	<0.001	1.213	0.952–1.546	0.117
Model 5^e^	0.556	0.361–0.858	0.008	2.058	1.455–2.911	<0.001	1.094	0.822–1.456	0.539
Model 6^f^	0.561	0.363–0.868	0.009	1.934	1.363–2.744	<0.001	1.043	0.781–1.393	0.775

^d^Original model without adjusting for any variables. ^e^Adjusted for age, gender, race, education level, marital status, income to poverty. ^f^Adjusted for the independent variables in Model 5 plus recent health status.

Based on the results of interaction analysis, to explore the effect of vitamin D deficiency on cognitive impairment at different levels of PA, we further divided PA into two subgroups, the high PA group and the low PA group, and within each of the two subgroups, we analyzed the effect of vitamin D deficiency on cognitive impairment screened by the three tests CERAD W-L/AFT/DSST. As in previous analyses, we set up the same three models (Models 4/5/6) to exclude confounding variables (age, gender, race, education level, marital status, income to poverty, and recent health status). The results showed that in AFT, Model 6 (excluded all confounders) showed: OR = 1.456, 95% CI: 1.067–1.986, *p* = 0.018 in the high PA subgroup; OR = 1.029, 95% CI: 0.792–1.337, *p* = 0.831 in the low PA subgroup. In DSST, Model 6 (excluded all confounders) showed: OR = 1.934, 95% CI: 1.363–2.744, *p* < 0.001 in the high PA subgroup; OR = 1.043, 95% CI: 0.781–1.393, *p* = 0.775 in the low PA subgroup. In CERAD W-L, statistical analysis was not meaningful, so we do not present the data. The results suggest that vitamin D deficiency is associated with higher odds of cognitive impairment in older adult population engaged in higher levels of PA, increasing their potential risk by 45.6% (AFT) and 93.4% (DSST). However, this association was not significant in the group of older adults engaged in lower levels of PA: statistically significant difference was not observed in the association of vitamin D deficiency with cognitive impairment (*p* > 0.05) (See Table 5).

## 4 Discussion

**Figure 4:**
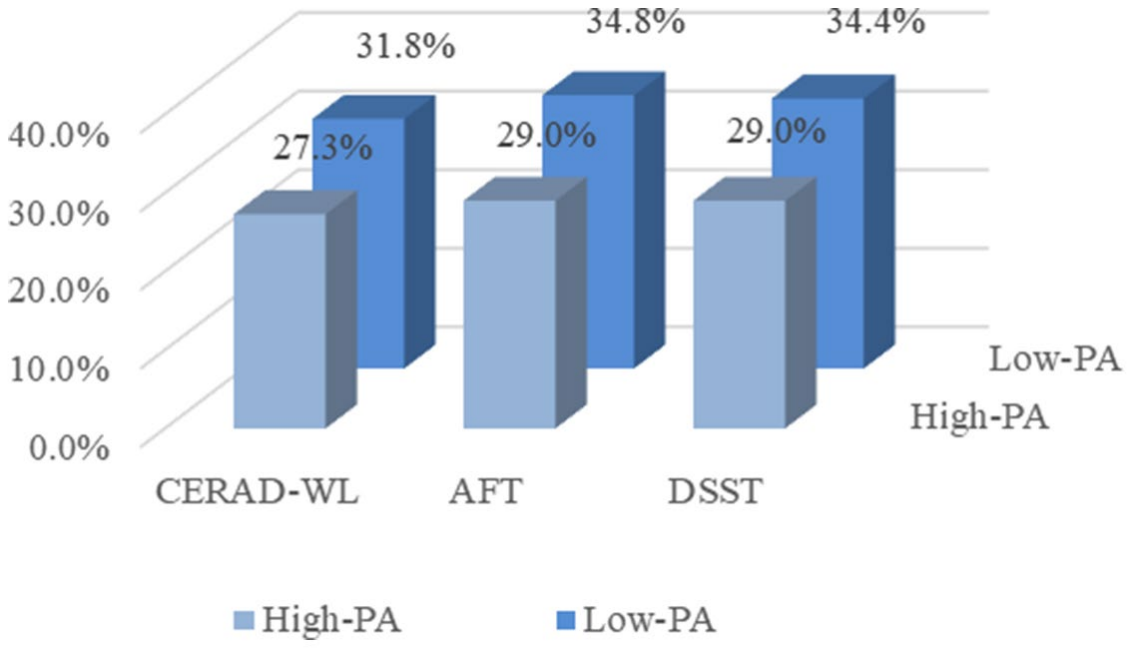
Proportion of three cognitive impairments in the presence of vitamin D deficiency at different levels of physical activity. CERAD-WL, Consortium to Establish a Registry for Alzheimer’s disease word learning test; AFT, Animal Fluency Test; DSST, Digit Symbol Substitution Test; and PA, Physical activity.

This study analyzed the effects of vitamin D and PA on cognitive function based on NHANES data from 2011 to 2014 and showed that vitamin D deficiency and low levels of PA may be associated with higher odds of cognitive impairment. There was a multiplicative interaction between the effects of vitamin D and PA on cognitive function, vitamin D deficiency is less prevalent in high levels of PA than in low levels of physical activity. The proportion of cognitive impairment in the three cognitive tests with vitamin D deficiency at high levels of PA was smaller than the proportion of cognitive impairment in the three cognitive tests with vitamin D deficiency at low levels of PA (Figure 4).

The present study suggests that men and women differ in the development of cognitive impairment and that the prevalence of cognitive impairment increases substantially with age. The differences in sex are caused by two factors, firstly an organizing effect, which occurs as early as during neuronal development, and secondly an activating effect, whereby sex steroids (e.g., estradiol) influence brain function in adulthood (22). Research by Hogervorst has shown that women who have menopause before the age of 47 have an increased risk of cognitive impairment in later life, suggesting that the risk of cognitive impairment increases with the number of years of hormone loss (23). Aging is a complex and irreversible process and is considered the most important risk factor for cognitive impairment. It occurs in multiple cellular systems and organs and is accompanied by a reduction in brain volume, loss of synapses, and enlargement of ventricles in specific regions of the brain, which may lead to cognitive decline (24).

Physical activity and vitamin D may differently affect various aspects of cognitive function, such as learning and memory, executive function, and attention and processing speed. PA supports memory and learning process by prompting the growth of new neurons, improving synaptic plasticity, and enhancing hippocampal function, which is critical for memory formation (25). Vitamin D is essential for synaptic plasticity, neurotransmission, and neuroprotection, therefore adequate vitamin D is important for cognitive function, including memory and learning ability (26). Executive functioning, including skills such as decision-making, problem-solving, and task-switching, can also be improved during engagement in PA (27), while maintaining optimal vitamin D levels can improve cognitive flexibility and problem-solving skills (28). PA also enhances neural connections and optimizes brain function, which leads to faster information processing and improved concentration and control (29). Research has shown that vitamin D deficiency is associated with slower processing speeds and reduced attention, thus maintaining optimal vitamin D levels may support these functions (30). Overall, PA and vitamin D have different effects on various aspects of cognitive function. Combining these factors through a healthy lifestyle that includes regular PA and maintaining optimal vitamin D levels may provide comprehensive support for cognitive health.

### 4.1 Differences in the results of subgroup analyses

The results of this study suggest that vitamin D deficiency is associated with higher odds of cognitive impairment at high levels of PA, but not at low levels of PA. There are several explanations for why vitamin D deficiency may affect cognitive function more strongly in individuals who participate in high PA. First, people who engage in high PA may not be spending more time outdoors. Evidence suggests that older adults tend to workout or exercise more in indoor settings (31, 32). Therefore, high PA sometimes did not increase their exposure to sunlight, which is the main way to obtain vitamin D (33). Additionally, high PA can place significant stress on the body, leading to inflammation and an increased demand for nutrients such as vitamin D (34). If an individual is deficient in vitamin D, the body may not be able to adequately repair the damage caused by the PA, leading to cognitive impairment over time. Another possible explanation for the link between vitamin D deficiency and cognitive impairment in individuals with high level PA is that this type of activity may place a greater cognitive demand on the brain. In order to perform complex movements and activities, individuals need to have good spatial awareness, attention, and executive function (35, 36). Vitamin D plays a crucial role in maintaining these cognitive functions, which may be why deficiency in this vitamin can be particularly damaging in individuals who engage in this type of physical activity. In contrast, low PA may not be as demanding on the body or brain, which could explain why vitamin D deficiency does not have a significant effect on cognitive function in this population.

However, it is important to note that this does not mean that older adults who engage in low PA do not need to be mindful of their vitamin D levels. In fact, even individuals who spend most of their time indoors or engage in only light PA can be at risk for vitamin D deficiency, particularly if they live in areas with limited sun exposure or have dietary restrictions that limit their intake of vitamin D-rich foods (37). Ultimately, the link between vitamin D deficiency and cognitive impairment is complex and multifaceted, and much more research is needed to fully understand how these two factors interact. However, maintaining adequate vitamin D levels as we age is critical to maintaining cognitive function, especially in individuals who engage in moderate to vigorous exercise. Previous studies have shown uncertainty about the potential effects of vitamin D supplementation on cognition (38, 39), so it is important for older adults at risk for vitamin D deficiency to work with their healthcare providers to determine the best course of action to maintain optimal vitamin D levels and minimize the risk of cognitive impairment.

### 4.2 Limitations

There are several limitations: (1) general limitations of NHANES cross-sectional studies. Including recall bias: NHANES cross-sectional study usually takes a question-and-answer format in the process of data collection, participants may have subjective or objective factors that may bias the results in the process of answering; Raw data covariates: there may be other influences on cognitive function that are not taken into account. Due to data limitations, some covariates were not included in this study, such as years of menopause. (2) Environmental-specific limitation: the study used NHANES data from 2011 to 2014, however, after the emergence of the Corona Virus Disease 2019 (COVID-19) pandemic may directly or indirectly lead to a decrease in the proportion of older adults in the total population and does not reflect the current situation well. (3) Limitations of nutritional factors: the vitamin D status involved in this study may be influenced by various nutritional factors such as diet, dietary habits, and fasting, which we did not include as covariates. In our future design, we will consider avoiding these limitations and further explore the deeper mechanics of PA and vitamin D on cognitive function.

## 5 Conclusion

The present study showed that low PA and vitamin D deficiency are both risk factors for cognitive impairment in elderly population, and low PA is also a risk factor for vitamin D deficiency. The study also showed an interaction between PA and vitamin D in their association with cognitive impairment. In older adults who participate in higher levels of PA, vitamin D deficiency is a risk factor for cognitive impairment; this association is not significant in older adults who engage in lower levels of PA. We advocated that older adults to participate in high levels of PA and take appropriate vitamin D supplements, to prevent cognitive impairment.

## Data availability statement

The raw data supporting the conclusions of this article will be made available by the authors, without undue reservation.

## Ethics statement

The studies involving humans were approved by National Center for Health Statistics (NCHS) Ethics Review Board. The studies were conducted in accordance with the local legislation and institutional requirements. The participants provided their written informed consent to participate in this study.

## Author contributions

JG: Conceptualization, Data curation, Formal analysis, Methodology, Project administration, Software, Validation, Visualization, Writing – original draft, Writing – review & editing. HM: Conceptualization, Data curation, Formal analysis, Investigation, Methodology, Resources, Supervision, Validation, Writing – review & editing. LZ: Conceptualization, Data curation, Software, Writing – review & editing. XZ: Funding acquisition, Supervision, Writing – review & editing.

## Glossary

WHO: World Health Organization
PA: Physical activity
MET: Metabolic equivalent
MVPA: Moderate-to-vigorous physical activity
NHANES: National Health and Nutrition Examination Survey
U.S.: The United States
NCHS: National Center for Health Statistics
CERAD: Consortium to Establish a Registry for Alzheimer’s disease
CERAD W-L: Consortium to Establish a Registry for Alzheimer’s disease Word Learning subtest
AFT: Animal Fluency test
DSST: Digit Symbol Substitution test
GPAQ: Global Physical Activity Questionnaire
CAPI: Computer Assisted Personal Interview
WPA: Work physical activity
TPA: Transportation physical activity
RPA: Recreational physical activity
CDC: Centers for Disease Control
HPLC-MS/MS: High-performance liquid chromatography tandem mass spectrometry
25OHD3: 25-hydroxyvitamin D3
epi-25OHD3: 3-epi-25-hydroxyvitamin D3
25OHD2: 25-hydroxyvitamin D2
SPSS: Statistical Package for Social Sciences
COVID-19: Corona Virus Disease 2019

## References

[1] Alzheimer's Disease International (2022). World Alzheimer Report 2022: Life after diagnosis: Navigating treatment, care and support. World Alzheimer Reports|Alzheimer's Disease International (ADI) (https://www.alzint.org/).

[2] Vega JN Newhouse PA. Mild cognitive impairment: diagnosis, longitudinal course, and emerging treatments. Curr Psychiatry Rep. (2014) 16:490. doi: 10.1007/s11920-014-0490-8 25160795 PMC4169219

[3] Jongsiriyanyong S Limpawattana P. Mild cognitive impairment in clinical practice: a review article. Am J Alzheimers Dis Other Dement. (2018) 33:500–7. doi: 10.1177/1533317518791401 PMC10852498 30068225

[4] World Health Organization (2012). Dementia: a public health priority. World Health Organization, Geneva, Switzerland. Retrieved from Dementia: a public health priority (https://www.who.int/).

[5] Landry GJ Liu-Ambrose T. Buying time: a rationale for examining the use of circadian rhythm and sleep interventions to delay progression of mild cognitive impairment to Alzheimer's disease. Front Aging Neurosci. (2014) 6:325. doi: 10.3389/fnagi.2014.00325 25538616 PMC4259166

[6] Dominguez LJ Veronese N Vernuccio L Catanese G Inzerillo F Salemi G. Nutrition, physical activity, and other lifestyle factors in the prevention of cognitive decline and dementia. Nutrients. (2021) 13:4080. doi: 10.3390/nu13114080 34836334 PMC8624903

[7] Ainsworth BE Haskell WL Herrmann SD Meckes N Bassett DR Jr Tudor-Locke C. 2011 compendium of physical activities: a second update of codes and MET values. Med Sci Sports Exerc. (2011) 43:1575–81. doi: 10.1249/MSS.0b013e31821ece12 21681120

[8] Hamer M Chida Y. Physical activity and risk of neurodegenerative disease: a systematic review of prospective evidence. Psychol Med. (2009) 39:3–11. doi: 10.1017/S0033291708003681 18570697

[9] Holick MF. The vitamin D deficiency pandemic: approaches for diagnosis, treatment and prevention. Rev Endocr Metab Disord. (2017) 18:153–65. doi: 10.1007/s11154-017-9424-1 28516265

[10] Gáll Z Székely O. Role of vitamin D in cognitive dysfunction: new molecular concepts and discrepancies between animal and human findings. Nutrients. (2021) 13:3672. doi: 10.3390/nu13113672 34835929 PMC8620681

[11] Sahota O. Understanding vitamin D deficiency. Age Ageing. (2014) 43:589–91. doi: 10.1093/ageing/afu104 25074537 PMC4143492

[12] Guzek D Kołota A Lachowicz K Skolmowska D Stachoń M Głąbska D. Association between vitamin D supplementation and mental health in healthy adults: a systematic review. J Clin Med. (2021) 10:5156. doi: 10.3390/jcm10215156 34768677 PMC8584834

[13] Annweiler C. Vitamin D in dementia prevention. Ann N Y Acad Sci. (2016) 1367:57–63. doi: 10.1111/nyas.13058 27116242

[14] Morris JC Heyman A Mohs RC Hughes JP van Belle G Fillenbaum G. The consortium to establish a registry for Alzheimer's disease (CERAD). Part I. Clinical and neuropsychological assessment of Alzheimer's disease. Neurology. (1989) 39:1159–65. doi: 10.1212/wnl.39.9.1159 2771064

[15] Strauss E Sherman EMS Spreen O. A Compendium of Neuropsychological Tests: Administration, Norms and Commentary. 3rd ed. New York: Oxford University Press (2006).

[16] Wechsler D. WAIS Manual. 3rd ed. New York: Psychological Corporation (1997).

[17] Dong X Li S Sun J Li Y Zhang D. Association of Coffee, decaffeinated coffee and caffeine intake from coffee with cognitive performance in older adults: National Health and nutrition examination survey (NHANES) 2011-2014. Nutrients. (2020) 12:840. doi: 10.3390/nu12030840 32245123 PMC7146118

[18] U.S. Department of Health and Human Services (2018). Physical activity guidelines for Americans. Current Guidelines Available at: https://health.gov

[19] Moorman JE Akinbami LJ Bailey CM Zahran HS King ME Johnson CA. National surveillance of asthma: United States, 2001-2010. Vital Health Stat. (2012) 3:1–58. 24252609

[20] Malik VS Willett WC Hu FB. Global obesity: trends, risk factors and policy implications. Nat Rev Endocrinol. (2013) 9:13–27. doi: 10.1038/nrendo.2012.199 23165161

[21] Gale SA Acar D Daffner KR. Dementia. Am J Med. (2018) 131:1161–9. doi: 10.1016/j.amjmed.2018.01.022 29425707

[22] Li R Singh M. Sex differences in cognitive impairment and Alzheimer's disease. Front Neuroendocrinol. (2014) 35:385–403. doi: 10.1016/j.yfrne.2014.01.002 24434111 PMC4087048

[23] Hogervorst E. Effects of gonadal hormones on cognitive behaviour in elderly men and women. J Neuroendocrinol. (2013) 25:1182–95. doi: 10.1111/jne.12080 23895362

[24] Breijyeh Z Karaman R. Comprehensive review on Alzheimer's disease: causes and treatment. Molecules. (2020) 25:5789. doi: 10.3390/molecules25245789 33302541 PMC7764106

[25] Voss MW Soto C Yoo S Sodoma M Vivar C van Praag H. Exercise and hippocampal memory systems. Trends Cogn Sci. (2019) 23:318–33. doi: 10.1016/j.tics.2019.01.006 30777641 PMC6422697

[26] Bivona G Lo Sasso B Gambino CM Giglio RV Scazzone C Agnello L. The role of vitamin D as a biomarker in Alzheimer's disease. Brain Sci. (2021) 11:334. doi: 10.3390/brainsci11030334 33800891 PMC8000099

[27] Zeng Y Wang J Cai X Zhang X Zhang J Peng M. Effects of physical activity interventions on executive function in older adults with dementia: a meta-analysis of randomized controlled trials. Geriatr Nurs. (2023) 51:369–77. doi: 10.1016/j.gerinurse.2023.04.012 37127013

[28] Annweiler C Montero-Odasso M Llewellyn DJ Richard-Devantoy S Duque G Beauchet O. Meta-analysis of memory and executive dysfunctions in relation to vitamin D. J Alzheimers Dis. (2013) 37:147–71. doi: 10.3233/JAD-130452 23948884

[29] Frederiksen KS Verdelho A Madureira S Bäzner H O'Brien JT Fazekas F. On behalf of the LADIS study physical activity in the elderly is associated with improved executive function and processing speed: the LADIS study. Int J Geriatr Psychiatry. (2015) 30:744–50. doi: 10.1002/gps.4220 25363336

[30] Lipowski M Walczak-Kozłowska T Lipowska M Kortas J Antosiewicz J Falcioni G. Improvement of attention, executive functions, and processing speed in elderly women as a result of involvement in the Nordic walking training program and vitamin D supplementation. Nutrients. (2019) 11:1311. doi: 10.3390/nu11061311 31212617 PMC6628124

[31] Tudor-Locke C Craig CL Aoyagi Y Bell RC Croteau KA De Bourdeaudhuij I. How many steps/day are enough? For older adults and special populations. Int J Behav Nutr Phys Act. (2011) 8:80. doi: 10.1186/1479-5868-8-80 21798044 PMC3169444

[32] McAuley E Motl RW Morris KS Hu L Doerksen SE Elavsky S. Enhancing physical activity adherence and well-being in multiple sclerosis: a randomised controlled trial. Mult Scler. (2007) 13:652–9. doi: 10.1177/1352458506072188 17548446

[33] Wiciński M Adamkiewicz D Adamkiewicz M Śniegocki M Podhorecka M Szychta P. Impact of vitamin D on physical efficiency and exercise performance-a review. Nutrients. (2019) 11:2826. doi: 10.3390/nu11112826 31752277 PMC6893541

[34] Madden RF Shearer J Legg D Parnell JA. Evaluation of dietary supplement use in wheelchair Rugby athletes. Nutrients. (2018) 10:1958. doi: 10.3390/nu10121958 30544913 PMC6315401

[35] Hötting K Röder B. Beneficial effects of physical exercise on neuroplasticity and cognition. Neurosci Biobehav Rev. (2013) 37:2243–2257. doi: 10.1016/j.neubiorev.2013.04.005 23623982

[36] Colcombe S Kramer AF. Fitness effects on the cognitive function of older adults: a meta-analytic study. Psychol Sci. (2003) 14:125–30. doi: 10.1111/1467-9280.t01-1-01430 12661673

[37] Palacios C Gonzalez L. Is vitamin D deficiency a major global public health problem? J Steroid Biochem Mol Biol. (2014) 144:138–45. doi: 10.1016/j.jsbmb.2013.11.003 24239505 PMC4018438

[38] Gil Martínez V Avedillo Salas A Santander BS. Vitamin supplementation and dementia: a systematic review. Nutrients. (2022) 14:1033. doi: 10.3390/nu14051033 35268010 PMC8912288

[39] Montero-Odasso M Zou G Speechley M Almeida QJ Liu-Ambrose T Middleton LE. Effects of exercise alone or combined with cognitive training and vitamin D supplementation to improve cognition in adults with mild cognitive impairment: a randomized clinical trial. JAMA Netw Open. (2023) 6:e2324465. doi: 10.1001/jamanetworkopen.2023.24465 37471089 PMC10359965

